# Modified iron phosphate/polyvinyl alcohol composite film for controlled-release fertilisers†

**DOI:** 10.1039/c8ra01843j

**Published:** 2018-05-17

**Authors:** Yi Zhang, Zhifeng Yi, Lianmei Wei, Lingxue Kong, Lijun Wang

**Affiliations:** School of Environmental and Materials Engineering, College of Engineering, Shanghai Polytechnic University Shanghai 201209 P. R. China ljwang@sspu.edu.cn; Deakin University, Institute for Frontier Materials Geelong Campus at Waurn Ponds Geelong Victoria 3216 Australia lingxue.kong@deakin.edu.au; Shanghai Innovation Institute for Materials Shanghai 200444 P. R. China

## Abstract

Traditional soluble phosphorous (P) fertilisers can be easily leached to pollute water systems, resulting in water eutrophication, a major environmental problem from the oversupply of unused nutrients. One innovative solution is to control the release of P upon demands of the plants. This study established a new concept of controlled-release P fertiliser *via* incorporation of ferric phosphate (FePO_4_) as a P source in polyvinyl alcohol (PVA) films, which can immobilise the FePO_4_ particles and stimuli-responsively accelerate their release rate in the presence of citric acid. More importantly, FePO_4_ used in this work originated from steelmaking slag as a potential waste reuse. Due to the low solubility of FePO_4_, diethylamine was introduced to modify FePO_4_ particles to facilitate the release of P before incorporating with PVA. The effects of diethylamine modification and the properties of FePO_4_/PVA films were systematically investigated through microscopic and spectroscopic methods. The release of P from particles and films was examined in both deionised water and citric acid solution for 30 days. The results showed a tenfold increase of the release rate of modified FePO_4_/PVA in citric acid solution compared with that in deionised water, and also a doubled release rate of the modified FePO_4_/PVA compared to that of FePO_4_/PVA in citric acid. The improved performance suggests that PVA can maintain the phosphorous content with exposure to water and expedite release in citric acid upon the demand of plants. This composite film offers a new opportunity for the application of insoluble phosphate as a phosphorous fertiliser.

## Abstract 2

A new concept of citric acid-stimulus P fertiliser *via* incorporation of ferric phosphate as P source in polyvinyl alcohol films.

## Graphical abstract

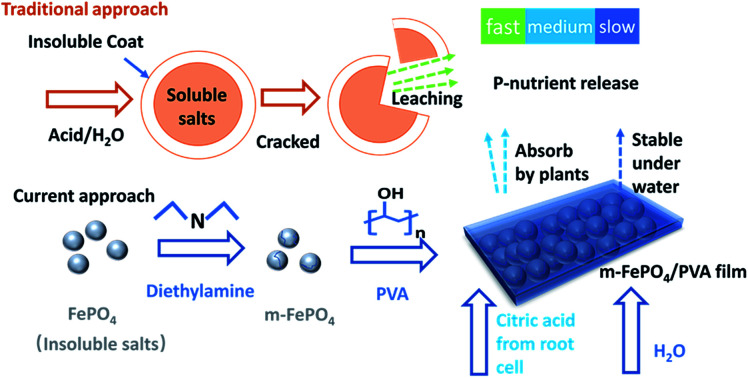

## Introduction

Phosphorous (P) as one of the most commonly used nutrients in agriculture can improve the nourishment of plants. Conventional soluble P fertilisers are made by dissolving phosphate rock (insoluble) in sulfuric acid^1–3^ to convert P into a soluble form (hydrogen phosphate and dihydrogen phosphate)^4^ which can be dissolved rapidly in water. However, soluble phosphate fertiliser (P-fertiliser) can be easily leached to pollute rivers and lakes, leading to water eutrophication and subsequent environmental issues.^5^ The overdose of P-fertiliser may also cause fertiliser burn to plants. Therefore, a controlled-release formulation of P-fertiliser is required to provide this nutrient in a controllable fashion to minimize environmental and economic risks.

Due to limitations of soluble phosphate fertilisers, the use of controlled-release fertiliser has become a promising direction to improve and manage P release.^6,7^ A traditional controlled-release formulation contains a soluble phosphate core as the source of nutrient and a relatively insoluble shell as a release controlling buffer. The shell is commonly fabricated using sulphur^8^ and polymer (*e.g.*, polyolefin, polyvinyl chloride (PVC), rubber, *etc.*). There are several problems with a sulphur-based shell. First, using sulphur as a shell may increase the acidity of soil, leading to calcium deficiency.^9^ Second, the mechanical property of a sulphur shell is poor for withstanding mechanical pressure during the fabricating or transporting processes. Third, once the sulphur shell is cracked by high osmotic pressure resulting from water absorption, all the loaded soluble fertiliser may be released instantly (catastrophic-type release) and the rate cannot be controlled.^10^ Although polymer coatings show an outstanding swelling property, degradation of a polymer, such as PVC and rubber, is a major disadvantage in applications of this type of controlled release system.^11^

In addition to the soluble fertiliser system, using insoluble salts like phosphate rock can offer a more controllable release rate and protection of phosphorous than soluble salts, as the release of phosphate is highly related to the solubility of a specific salt. Recently, ferric phosphate (FePO_4_) has been widely used to replace scarce mineral sources such as phosphate rock, as it can be recycled from the by-products from steelmaking slag in the steel making industry, indicating a great environmental sustainability.^12^ Besides the nutrient providing function, FePO_4_ can also eliminate snails and slugs *via* interfering their metabolic systems. However, the low solubility of FePO_4_ (0.6 g/100 mL at 100 °C)^13^ may limit its direct application in agriculture. Approaches have been developed to increase the availability of P and facilitate its release. Zhong *et al.*^14^ embedded phosphate rock in sulfonated corn starch to achieve a release rate of 4.5 times more than the raw phosphate rock, but the sulfonate modification method may lead to soil acidulation. In addition, increasing the surface area may also improve the dissolution of insoluble salts as the result of an increase in available area for solvation.^15^ Therefore, an agent that can modify the surface of the insoluble particles leading to a larger surface area is desired. Diethylamine (DEA) is one of the modification agents that have been used in catalysis to increase their surface areas,^16^ and thus can be a potential modifier for FePO_4_. However, the application of DEA on FePO_4_ particles was merely investigated in the literature. Also, DEA can be toxic and corrosive if it is not well administered and its amount of use should be controlled within safety limits.

Washing off FePO_4_ powder as a fertiliser is still an issue to be dealt with. A film-based fertiliser system could potentially immobilise FePO_4_ powders. In this case, polyvinyl alcohol (PVA) can be an ideal candidate as the polymer matrix to be incorporated with the phosphate salt, because of the non-toxic, biodegradable^17^ and water-soluble^18^ properties of PVA. Due to its excellent film forming and high mechanical strength,^19,20^ PVA can be applied as a sizing, coating, and thickening agent to package powders away from running water invasion. PVA-composited film fertiliser can also be made into any shape and be easily sustained in the soil. Han *et al.*^21^ prepared a controlled-release nitrogen fertiliser by coating a starch/PVA onto the soluble granules, and demonstrated that permeability of the NH_4_^+^ is related to the thickness of the PVA coating package. Also, Noppakundilograt *et al.*^22^ utilized PVA as a film former and as the first coating layer to form a 3-layer controlled-release fertiliser; the PVA-coated fertiliser displayed a 4.5 times slower rate than the uncoated one. Most recently, Sarkar *et al.*^23^ coated a partially acidulated phosphate rock with PVA. The above studies used PVA as a coating film to delay the dissolving of the soluble core; however, boosting PVA on release of an insoluble salt like FePO_4_ has yet to be investigated.

Herein, an approach to synthesise a new film-type phosphate fertiliser is proposed. FePO_4_ is employed as phosphate source while the PVA acts as an auxiliary matrix. The modification of FePO_4_ is examined by using different concentrations of the modifier. The morphology and structures of modified FePO_4_ (m-FePO_4_) and PVA composited film were systematically investigated. Release kinetics of m-FePO_4_/PVA fertiliser was carried out in deionised water as a control group and in solution of citric acid that mimics the environment around plant roots. This study will deliver a totally new platform for slag from industrial waste to be potentially used as P-fertiliser.

## Experimental section

### Materials

Polyvinyl alcohol (PVA) (*M*_W_ 146,000–186 000, 99 wt% hydrolysed), diethylamine (DEA) (99.5 wt%), l-ascorbic acid (reagent grade), ammonium molybdate (bioreagent 81.0–83.0% MoO_3_ basis), potassium antimony(iii) tartrate hydrate (99 wt%), citric acid (ACS reagent, ≥99.5%) and potassium dihydrogen phosphate (powder, 98 wt%) were purchased from Sigma-Aldrich (St. Louis, USA). Sulfuric acid (98 wt%) and hydrochloric acid (35 wt%) were purchased from Chemsupply (Adelaide, Australia). FePO_4_ (98 wt%) was refined from iron phosphate slag (82 wt%) which was described in our earlier work.^24^

### Modification of FePO_4_

FePO_4_ was modified by DEA in aqueous solution. Specifically, 0.1 g of FePO_4_ was dispersed in 50 mL of deionised water and sonicated for 5 min. After that, a group of five predetermined amounts of DEA (the ratio of DEA to FePO_4_ are 0.01 : 1 wt, 0.03 : 1 wt, 0.04 : 1 wt, 0.05 : 1 wt, and 0.1 : 1 wt, respectively) were added into the FePO_4_ suspension prior to being mixed in a tube roller mixer for 12 h. The modified FePO_4_ were centrifuged in 50 mL centrifuge tubes at 10 000 rpm for 5 min and washed twice with deionised water to remove residual DEA. The cleaned samples were concentrated and kept in deionised water for further use (concentration 250 mg mL^−1^).

### Preparation of m-FePO_4_/PVA composite fertiliser

**Fig. 1:**
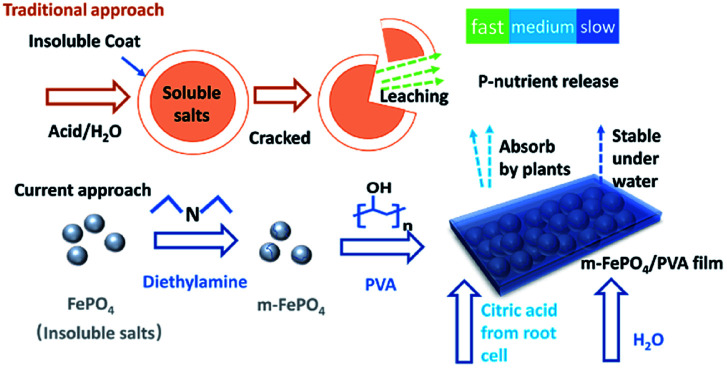
Schematic of traditional controlled-release fertiliser and fabrication of the current film-type P fertiliser.

The m-FePO_4_ derived from 0.03 : 1 wt of DEA to FePO_4_ ratio was selected as the best condition and a series of samples with different ratios between PVA and m-FePO_4_ were prepared. Specifically, 10.0 g of PVA was dissolved in 90.0 g of deionised water at 90 °C for 3 h under stirring. The suspension of m-FePO_4_ and PVA aqueous solution was mixed at m-FePO_4_/PVA (v/v) volume ratio of 3 : 1, 1 : 1, and 1 : 3, respectively (corresponding weight ratio of 75 : 1 wt, 25 : 1 wt, and 0.83 : 1 wt). The viscous mixture solution was further mixed on a Vortex Mixer (Ratek, Australia). The total volume of all the samples was kept at 4 mL. The m-FePO_4_/PVA mixtures were dropped (each droplet 0.2 mL) onto the surface of Parafilm® (Bemis, USA) which provided a hydrophobic surface for the formation of composite films. After drying for 24 h at room temperature, films with a diameter of about 0.5 cm were removed from the Parafilm® for further use. The procedure is shown in Fig. 1.

### Characterization

Scanning electron microscopy (SEM) samples were coated with a thin layer of carbon (powder samples) or gold (film samples) using an EM ACE600 sputter coater (Leica Microsystems, Wetzlar, Germany). SEM images were captured on a Supra 55VP (Zeiss, Oberkochen, Germany). The surface area of the samples was measured by a nitrogen adsorption instrument (ASAP 2020, Micromeritics, USA) at 77 K and calculated using the Brunauer–Emmett–Teller (BET) method. The cross-section of FePO_4_/PVA film was obtained by fracturing the samples in liquid nitrogen. Modified FePO_4_ powder was characterized by X-ray diffraction (XRD) (PANalytical, Netherlands) and the particle size was measured by Mastersizer (Malvern, UK). Attenuated total reflectance-Fourier-transform infrared spectroscopy (ATR-FTIR) technique was used to characterize the chemical structure of the modified FePO_4_ powder and FePO_4_/PVA films *via* a Bruker Vertex 70 spectrometer (Massachusetts, USA). The spectra were obtained at a resolution of 4 cm^−1^ in a range from 600 to 4000 cm^−1^. The percentage of FePO_4_ in FePO_4_/PVA film was confirmed by measuring absorbency with a USB-2000 UV-Vis (Ultraviolet and Visible Absorption Spectroscopy) spectrophotometer (Ocean Optics, Dunedin, FL, USA) after dissolving the samples in hydrochloric acid (35 wt%). A calibration curve of FePO_4_ was obtained through dissolving pure FePO_4_ (>99%) in hydrochloric acid and taking 334.49 nm as the absorption peak.

### Release of m-FePO_4_/PVA composite fertiliser in deionised water and in citric acid solution

The release behaviours of m-FePO_4_/PVA in deionised water and citric acid solution were examined. The content of FePO_4_ in all the samples were kept identical. The m-FePO_4_/PVA films were added into 15 mL centrifuge tubes with 2.5 mL of deionised water and 50 μmol mL^−1^ of citric acid solution, respectively. The release of P was monitored at room temperature and recorded for 30 days. On each day, 2 mL of the solution was removed to measure the concentration of phosphorous. After that, 2 mL of fresh deionised water or citric acid solution was added to maintain a constant volume. Phosphorus contents were estimated using colorimetric analysis.^25,26^

## Results and discussion

### Morphology of the m-FePO_4_

**Fig. 2:**
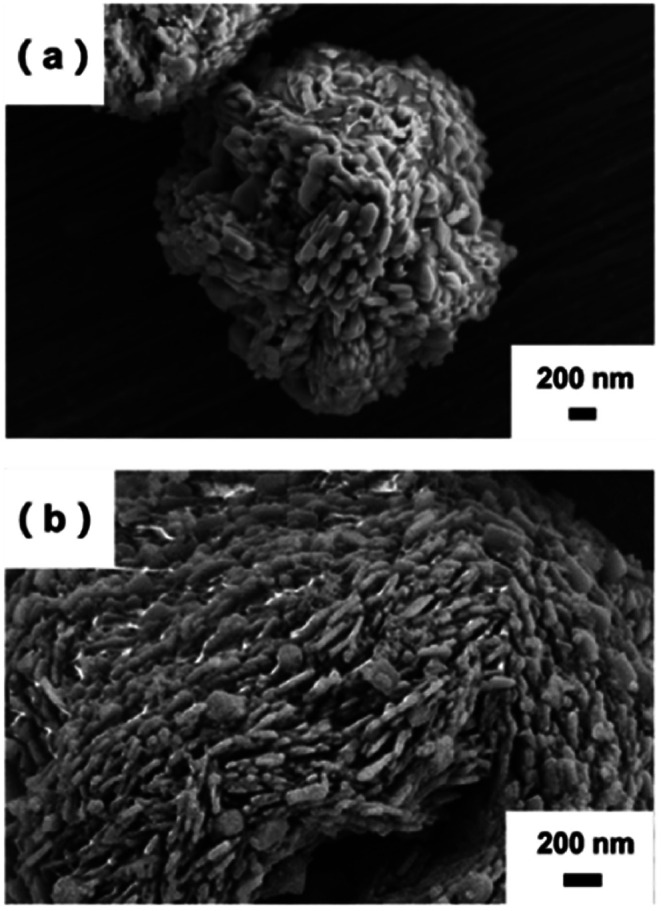
(a) The raw iron phosphate. (b) m-FePO_4_ resulting from the 0.03 : 1 wt of DEA to FePO_4_ ratio.

The morphology of the raw FePO_4_ particles is shown in Fig. 2a, while Fig. 2b and S1† illustrate the appearance of flake-structures on the surface of the m-FePO_4_. With increasing of DEA content, the flakes on the m-FePO_4_ particles become more noticeable (Fig. S1†). The surface areas of m-FePO_4_ treated with different amounts of DEA were further measured. It can be seen from Fig. S2a† that the surface area increases with the amount of DEA. This could be attributed to the flake structure as observed under SEM. An increased surface area could offer more contacts with water, leading to an expedited dissolution of phosphate during the release process.

The distribution of particle size with different DEA contents is shown in Fig. S3.† The majority of m-FePO_4_ particles have a diameter of around 50 μm. The volume fraction of small particles (diameters ranged from 0 to 25 μm) resulting from a small portion of DEA (from 0.01 : 1 wt to 0.05 : 1 wt of DEA to FePO_4_ ratio) does not show a noticeable change, while a remarkable increase was observed at a higher DEA content (the DEA to FePO_4_ ratio ranged from 0.05 : 1 wt to 0.1 : 1 wt). The increase in volume fraction of small particles could be due to the increased reaction of FePO_4_ with DEA, which will be further discussed in the following sections.

### Structure and composition of m-FePO_4_

**Fig. 3:**
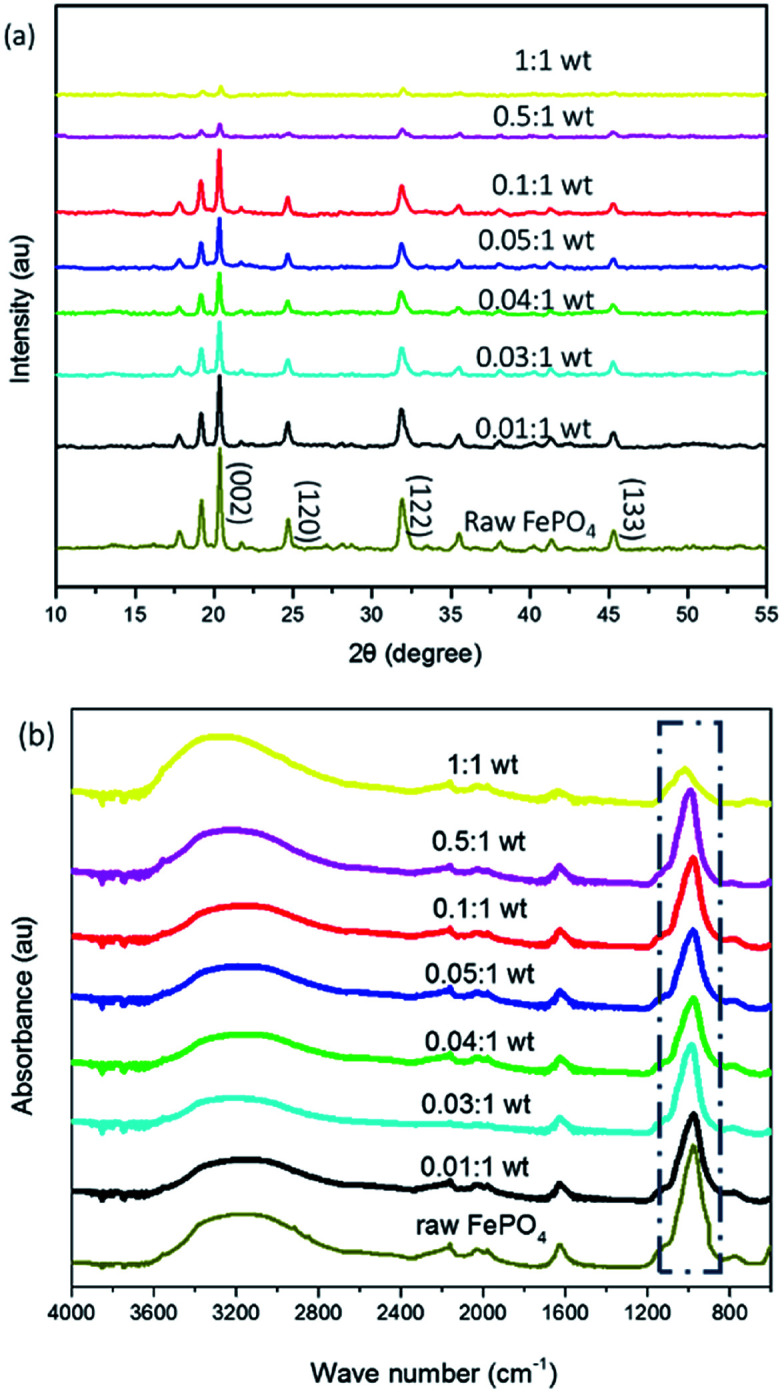
(a) XRD and (b) FTIR spectra of FePO_4_ particles treated with varied amounts of DEA after 24 h (the ratio on the curves denotes the ratio of the DEA to the raw FePO4 particles).

Fig. 3a shows the XRD patterns of samples which were modified with different amounts of DEA. The patterns were analysed using Jade software (Materials Data Inc, USA), using peaks at (002), (120), (122), and (133) (JCPDS card no. 33-0666) as the standard. With increasing amounts of DEA, the intensity of characteristic peaks (002) for the FePO_4_ decreased. This indicated that the crystalline structure of FePO_4_ gradually disappeared and more amorphous structures were formed.

In addition, FTIR was employed to examine changes of m-FePO_4_ with different contents of DEA. Fig. 3b shows the FTIR spectra of m-FePO_4_, which reveal bending and stretching vibrations of the phosphate group (PO_4_^3−^) at 800 to 1200 cm^−1^.^27^ The intensity of the peak at 1000 cm^−1^ declines when the content of DEA increases, thereby indicating the decrease of phosphate content remaining in the FePO_4_ particles with the addition of DEA. This is further confirmed by Fig. S2b.†

### Reaction between DEA and FePO_4_

A higher concentration of DEA (0.5 : 1 wt and 1 : 1 wt of DEA to FePO_4_ ratio) and a strong base (sodium hydroxide) was introduced to investigate the reaction between DEA and FePO_4_. It can be seen from Fig. S4† that the colour of the samples with 0.01 : 1 to 0.1 : 1 wt of DEA to FePO_4_ ratio gradually changed from white to brown, while the colour turned to dark red for the samples treated with high amounts of DEA (0.5 : 1 wt and 1 : 1 wt of DEA to FePO_4_ ratio). Similarly, the colour of dark red is observed with the adding of identical amounts of sodium hydroxide. The dark red colour is a characteristic indicator of the presence of Fe(OH)_3_. Furthermore, the P content in the supernatant after centrifugation was also measured and the results are shown in Fig. S2b,† which indicates that the dissolution of FePO_4_ produces PO_4_^3−^ in the solution and more likely precipitates Fe(OH)_3_ due to the large amount of OH^−^ in the solution. Therefore, the reaction between DEA and FePO_4_ is proposed as follows:1a
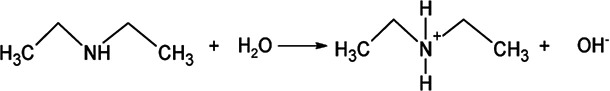

1b FePO_4_ + 3OH^−^ → Fe(OH)_3_ + PO_4_^3−^

Proposed reaction between FePO_4_ and DEA.

The formation of Fe(OH)_3_ is not desirable, as the PO_4_^3−^ was disposed of with the supernatant, leading to a decrease of the P content remaining in the particles. The obvious change of colour happened at the 0.04 : 1 of DEA to FePO_4_ ratio (Fig. S4†), therefore, the optimal ratio was selected at 0.03 : 1, which has a low phosphate loss and reasonable flake structure that provided a relative large contact area with water. In addition, the use of DEA at this ratio is 29 μg g^−1^ FePO_4_, which is much less than the toxicity limit of diethylamide (LD_50_ = 540 μg g^−1^).^28^ The following experiment was conducted with m-FePO_4_ resulting from this ratio. The film properties and the release behaviour were examined.

### Morphology and structure of m-FePO_4_/PVA films

**Fig. 4:**
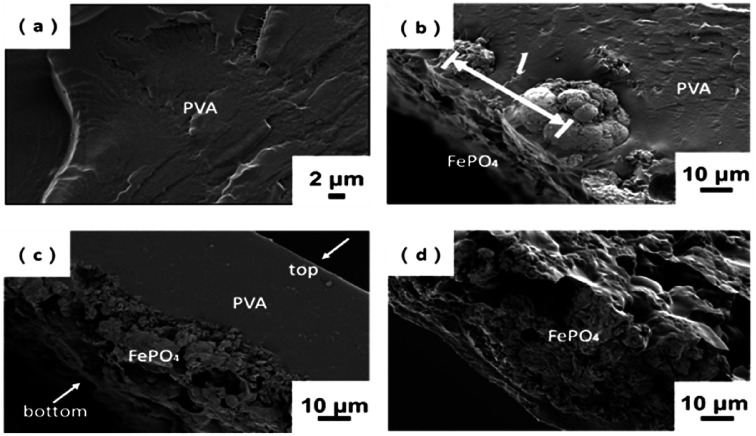
Cross-section of (a) raw PVA; (b) m-FePO_4_/PVA = 0.83 : 1 (w/w); (c) m-FePO_4_/PVA = 25 : 1 (w/w); and (d) m-FePO_4_/PVA = 75 : 1 (w/w).

**Fig. 5:**
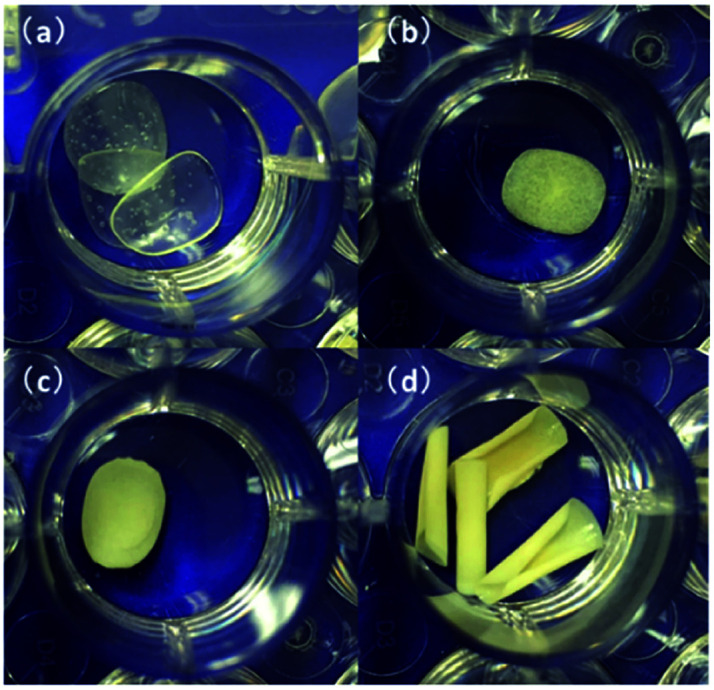
(a) Pure PVA films; (b) m-FePO_4_/PVA (w/w) = 0.83 : 1; (c) m-FePO_4_/PVA (w/w) = 25 : 1; and (d) m-FePO_4_/PVA (w/w) = 75 : 1.

Fig. 4 shows morphology of the cross-section of m-FePO_4_/PVA composite films. The majority of the m-FePO_4_ particles were distributed at the bottom of the PVA matrix as the particle size of m-FePO_4_ was still at a micron scale and the precipitation rate was very high. The PVA matrix shows a uniform one phase without any pores. The sediment of m-FePO_4_ on one surface is beneficial for the dissolution process and the release of phosphorous, as a prior swelling and degradation of dense PVA film is not required. PVA in this case was used as a matrix to hold all m-FePO_4_ particles in the film (Fig. 5), which can protect the nutrient from being washed away compared to a powder- or granules-based fertiliser system.

**Fig. 6:**
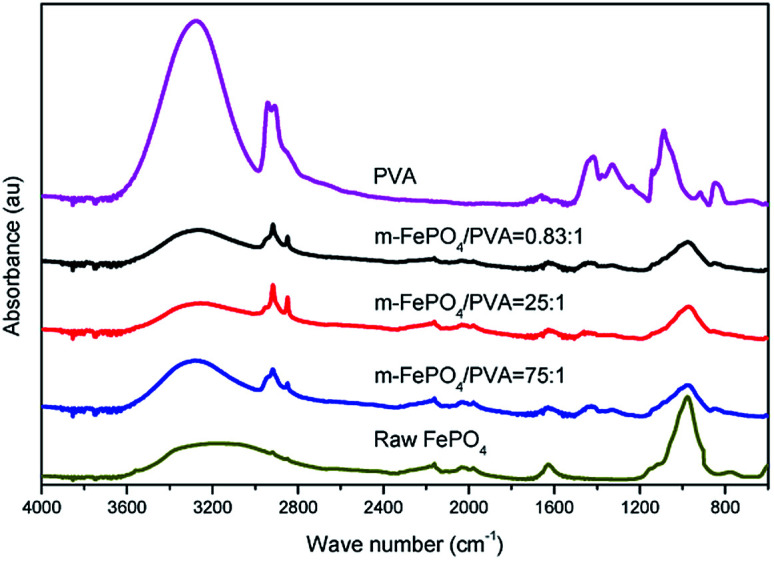
FTIR of m-FePO_4_/PVA with different mixture ratios.

The composition of m-FePO_4_/PVA films was confirmed by FTIR (Fig. 6). The absorption peaks at around 800–1200 cm^−1^ are regarded as P–O bonds.^29,30^ The peak at around 3640–3100 cm^−1^ represents O–H from inter- and intra-molecular hydrogen bonds,^31^ while the peak at 1740 cm^−1^ corresponds to the C=O bonds. Characteristic peaks from phosphate and PVA are present in all samples, so it may be concluded that the chemical structures of PVA and FePO_4_ were retained in the composite film.

### Release behaviours of m-FePO_4_/PVA in water and citric acid

When plants lack phosphorous they release organic acids, such as oxalic acid, malic acid, and citric acid.^32^ In this study, citric acid was selected as a typical organic acid generated by a plant to investigate the release behaviour of powder samples and our film-type fertiliser. The concentration of citric acid used was 50 μmol mL^−1^ which is the concentration normally produced by plant roots when phosphorus deficiency happens.^33^

**Fig. 7:**
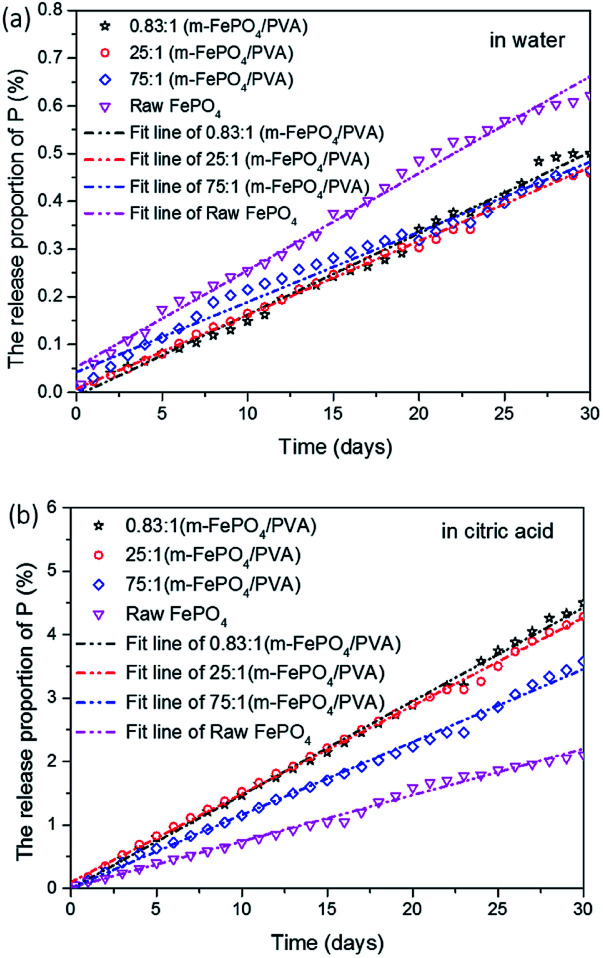
(a) The release proportion of phosphate in water. (b) The release proportion of phosphate in citric acid solution.

The release behaviour of film-type fertilisers with different ratios of m-FePO_4_ and PVA was investigated in citric acid and in water and the results are shown in Fig. 7a and b. All samples displayed linear release kinetics which was fitted with a linear release model. The equation is expressed as follows:*Y* = *Kx* + *b*where *Y* is the amount released in percentage, *x* is the time, *K* is the kinetic constant, and *b* is the intercept. The calculated parameters are listed in Table 1. *R*^2^ represents the accuracy of the fittings, which are all higher than 0.99, indicating a good linear regression. By comparing *K* values for all the samples, all samples showed a faster release rate in citric acid solution than in water. The increase of m-FePO_4_ content did not significantly affect the release rate in water and citric acid solution. The m-FePO_4_ to PVA ratios of 0.83 : 1 and 25 : 1 possess the lowest release rates in deionised water, while having the highest release rate in citric acid solution (90% faster than in water). This may be attributed to the increase of free PVA area that expedites the P release in the citric acid but hinders the release rate in water. These results indicate that the current film-type fertiliser can maintain P content in not only a water environment but stimuli-responsively accelerates the release amount of P in the presence of citric acid.

**Table 1:** Calculated parameters of the release kinetics for all the samples

Samples (m-FePO_4_/PVA)	*K*	*b*	*R* ^2^	Life (year)
0.83 : 1 in water	0.016	0.037	0.990	17.3
25 : 1 in water	0.016	0.002	0.997	17.6
75 : 1 in water	0.016	0.007	0.999	17.4
Raw FePO_4_ in deionised water	0.021	0.029	0.993	13.3
0.83 : 1 in citric acid	0.138	0.148	0.994	2.0
25 : 1 in citric acid	0.138	0.112	0.998	2.0
75 : 1 in citric acid	0.115	0.007	0.996	2.4
Raw FePO_4_ in citric acid solution	0.073	−0.129	0.992	3.8

Based on the kinetic model proposed, the life cycle of the current m-FePO_4_/PVA can be predicted. At 100% released phosphate, the calculated life cycle of the fertiliser is listed in Table 1. Assuming a constant presence of the citric acid, the estimated life cycle of film composited at three different ratios of m-FePO_4_ to the PVA contents, 0.83 : 1, 25 : 1, and 75 : 1, is 2.0, 2.0, and 2.4 years, respectively, which all are less than the raw FePO_4_ powder (3.8 years). In contrast, the life cycle of the film in water only is around 17 years, which is more than that of raw FePO_4_ powder (13.3 years). These results further suggest that the incorporation of PVA can control the release of phosphate, namely maintaining P content in the absence of organic acid but also facilitating the release of phosphate in the presence of an organic acid.

In general, the total amount of P required to grow a plant depends on the total weight of it, as described by grams of P per fresh weight of plant (g kg^−1^).^34^ The P requirements of different plant species and different plant growth cycles usually are varied, which makes it difficult to determine the appropriate P weight for plants. However, a standard medium can provide a reference to roughly determine the appropriate P content range. The safe concentration range of P in the Murashige and Skoog (MS) medium, as a common medium for the *in planta* nutrient experiments, is between 6 mmol L^−1^ (lower bound) and 1 mol L^−1^ (upper bound).^35^ When comparing this with the daily release of the current film-type fertiliser, the released phosphate ranges from 0.01 to 0.011 mol L^−1^ in water and 0.079 to 0.11 mol L^−1^ in citric acid solution, which is much more than the lower bound of P concentration in the MS medium. The release amount of P in both water and citric acid solution can meet the requirement for plant growth to some extent. Also, the release rate can be finely tuned by tailoring the ratio between m-FePO_4_ and PVA for specific plant species.

**Fig. 8:**
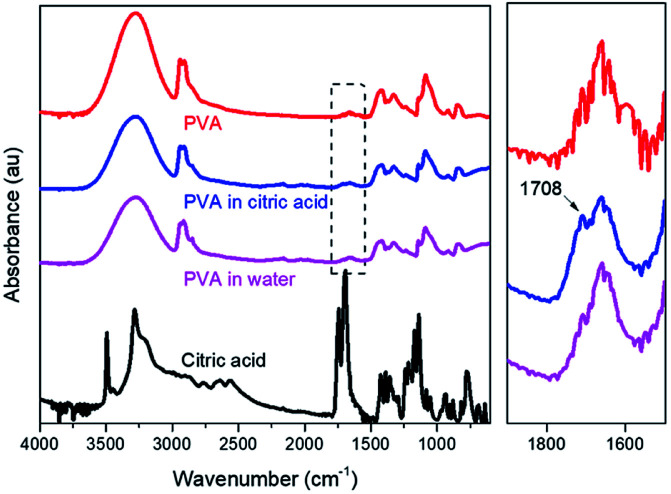
FTIR of PVA in deionised water and citric acid.

The PVA films after a 30 day release experiment were washed and dried for FTIR measurements (Fig. 8). The results show that there is a new peak present at around 1708 cm^−1^ in the sample immersed in citric acid solution, which corresponds to the carboxylic groups. Citric acid containing both hydroxyl and carboxyl groups hydrogen bonding can occur even at a low temperature (5 °C^36^); thus, combined with FTIR results the citric acid is more likely to be adsorbed on PVA, which has indirectly increased the density of acidity just around the surface of the film, facilitating the release of phosphate.

**Fig. 9:**
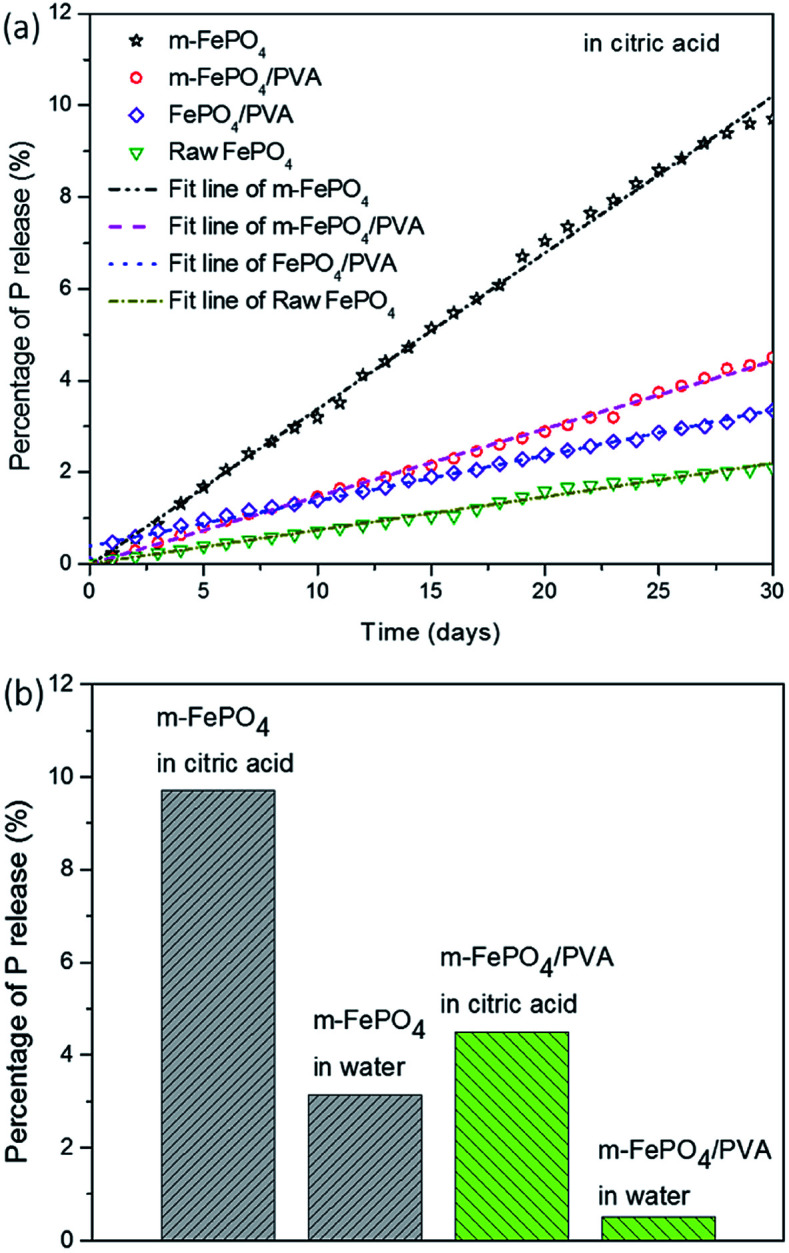
(a) P release of m-FePO_4_, m-FePO_4_/PVA, FePO_4_, and FePO_4_/PVA in the citric acid. (b) P release of the particle-type fertiliser (m-FePO_4_) and the film-type fertiliser (m-FePO_4_/PVA) in water and in citric acid solution.

Fig. 9a presents the results of P release from four types of fertiliser in citric acid solution for 30 days. The sample of m-FePO_4_ powders has the highest release rate while the raw FePO_4_ powders release at the lowest rate. This may be due to an increased surface area that benefits from the contact between particles and solution, leading to a faster release of P. It is interesting to note that PVA moderates the release of m-FePO_4_ while accelerating P release in the raw FePO_4_. This can be explained as that the m-FePO_4_ has a higher surface area to be integrated with PVA than the raw one, so the area of exposed PVA that contributes to the adsorption of citric acid is much less than raw one. Therefore, the PVA covers one side of the m-FePO_4_ particles to reduce the valid surface area while acting as a citric acid adsorption container to facilitate P release.

Fig. 9b shows the final release amount of P after 30 days. The P release in citric acid solution is greater than that in the water counterpart for both the particle-type fertiliser and the film-type fertiliser. A tenfold increase of the release rate of m-FePO_4_/PVA was found in citric acid solution than in the deionised water. The release rate of the m-FePO_4_ is twofold when compared to the raw one in the citric acid. Even though m-FePO_4_ powder possesses a higher release amount and rate than FePO_4_/PVA, powder fertilizer is more easily washed away than the film-type during irrigation, which indicates the advantage of the film-type fertilizer developed in this paper.

## Conclusions

A formulation of controlled-release phosphorus fertiliser was fabricated using recycled FePO_4_ as the P source and PVA as the polymeric matrix to form a freestanding film. The modification of FePO_4_, aiming at increasing the surface area of the particles, was examined in terms of the effect of DEA concentration, structure, and reaction mechanism. PVA acts as matrix to keep the particles from water washing away. It was found that this new film-type fertiliser is responsive to an organic acid, such as citric acid. The release rate of m-FePO_4_/PVA film is slower than that of raw FePO_4_ powder in deionised water, while it is faster in the presence of citric acid. This suggests that the phosphorous content can be maintained in water and released in citric acid solution such as that generated by plants when they have phosphorous deficiency. In addition, through manipulating the ratio between m-FePO_4_ and PVA, the release rate can be accurately tuned, which offers an opportunity to adapt for a specific condition of plants in agriculture. More importantly, FePO_4_ was recycled from steelmaking slag so the cost of other materials used in this work is low. This fabrication process can be easily scaled up at low cost, making it potentially suitable for large field use in agriculture. Except for the citric acid, the organisms in soil will also release other types of organic acid,^37^ on which the response of the film fertiliser will be further investigated in future work.

## Conflicts of interest

There are no conflicts to declare.

## Supplementary Material

RA-008-C8RA01843J-s001
