# Supplementary material for: Modified iron phosphate/polyvinyl alcohol composite film for controlled-release fertilisers
Source: RSC Adv. 2018 May 17;8(32):18146–52. doi: 10.1039/c8ra01843j (PMC9080590; doi:10.1039/c8ra01843j)
Supplement: RA-008-C8RA01843J-s001 [file RA-008-C8RA01843J-s001.pdf]

Electronic Supplementary Material (ESI) for RSC Advances.
This journal is © The Royal Society of Chemistry 2018

Supporting information

Modified Iron phosphate/Polyvinyl Alcohol Composite Film for Controlled-release Fertilisers

Yi Zhang[a, b], Zhifeng Yi[b], Lianmei Wei[a], Lingxue Kong[a, b, *], Lijun Wang[a, c*]

[a]School of Environmental and Materials Engineering, College of Engineering, Shanghai Polytechnic University, Shanghai, 201209, P. R. China

[b]Deakin University, Geelong, Institute for Frontier Materials, Geelong Campus at Waurn Ponds, Victoria 3216, Australia

[c]Shanghai Innovation Institute for Materials, Shanghai,200444, P. R. China

* Corresponding authors:

Lingxue Kong (Tel: +61 3 522 72087, Email: lingxue.kong@deakin.edu.au)

Lijun Wang (Tel: +86 021 5021 6696, Email: ljwang@sspu.edu.cn)

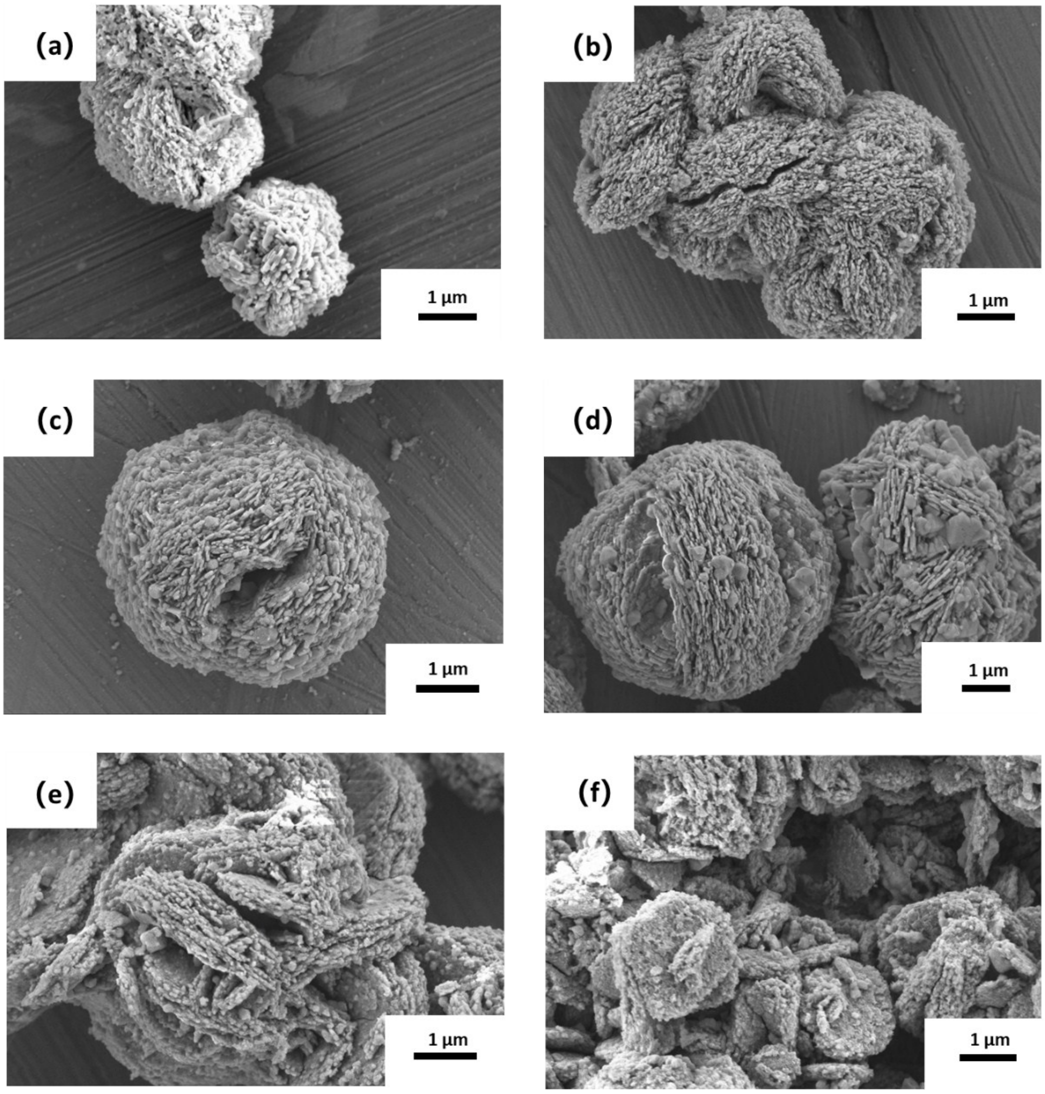

Figure S1 (a) The raw $FePO_4$ and m-$FePO_4$ treated with different diethylamine amount: (b) 0.01:1 wt , (c) 0.03:1 wt , (d) 0.04:1 wt, (e) 0.05:1 wt and (f) 0.1:1 wt.

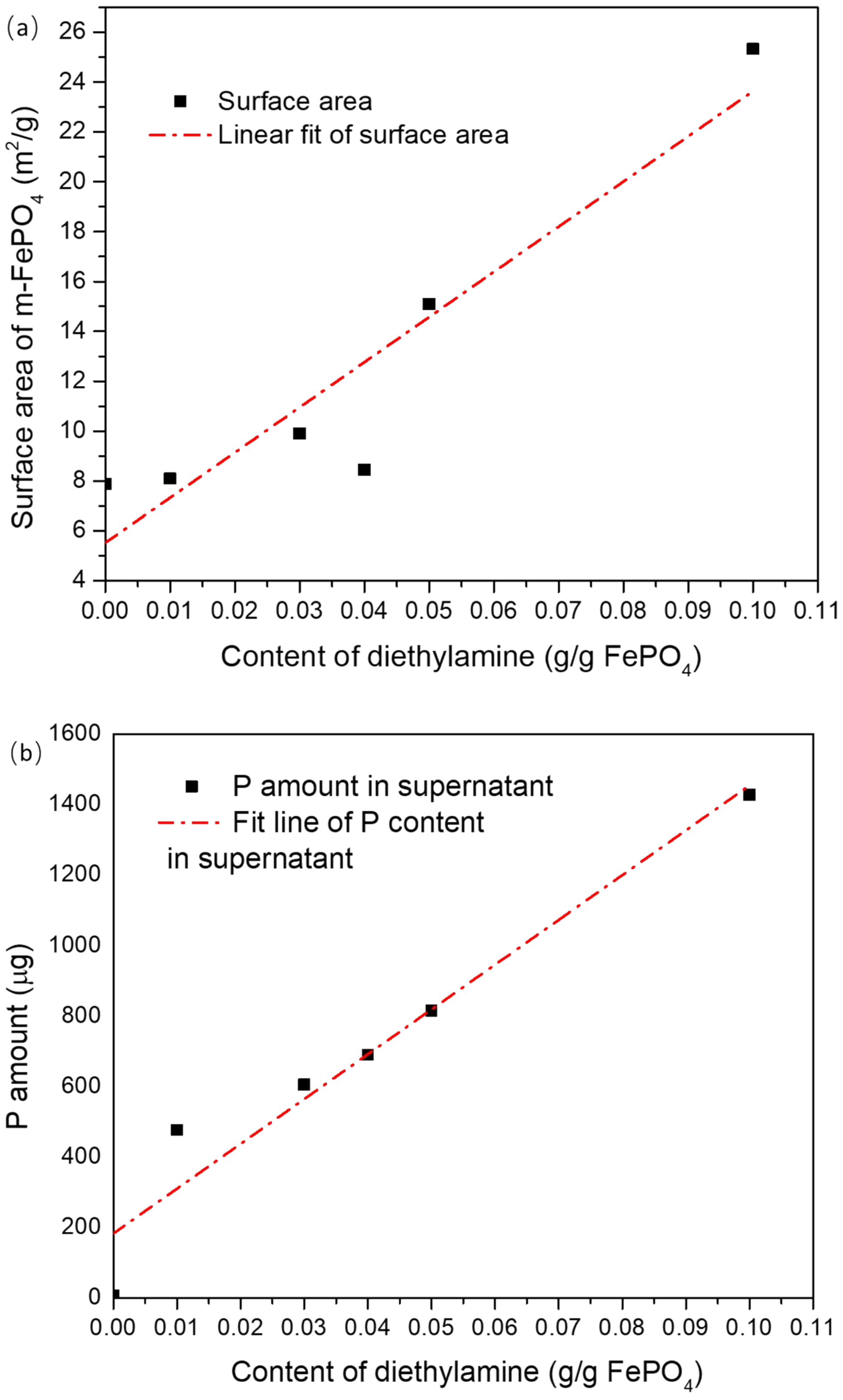

Figure S2 (a) Surface area and (b) P amount in supernatant of the raw $FePO_4$ and the m-$FePO_4$

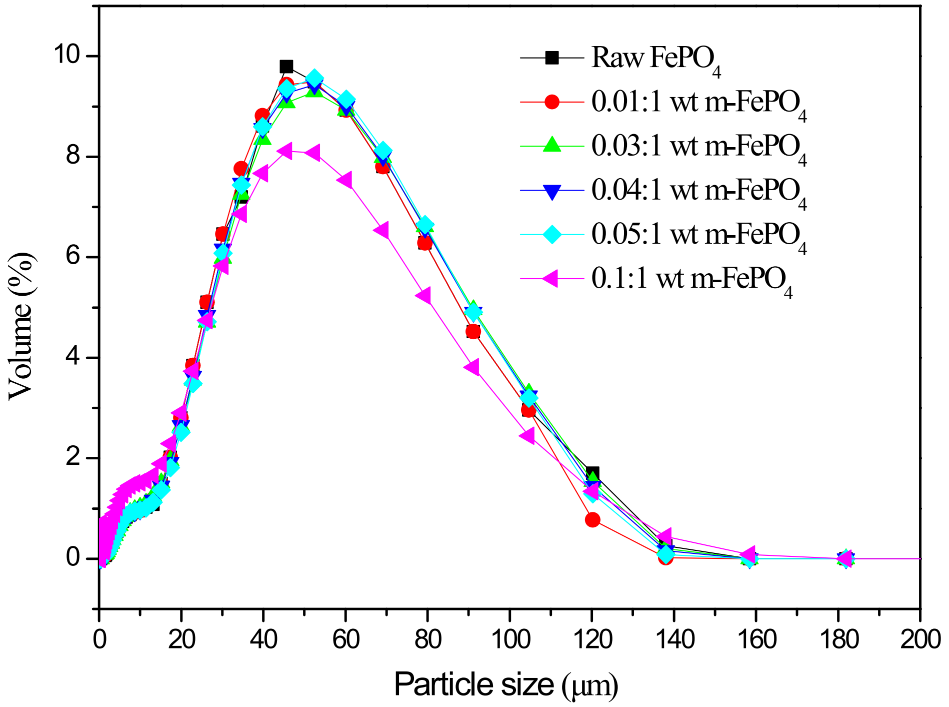

Figure S3 Particle size of raw $FePO_4$ and m-$FePO_4$

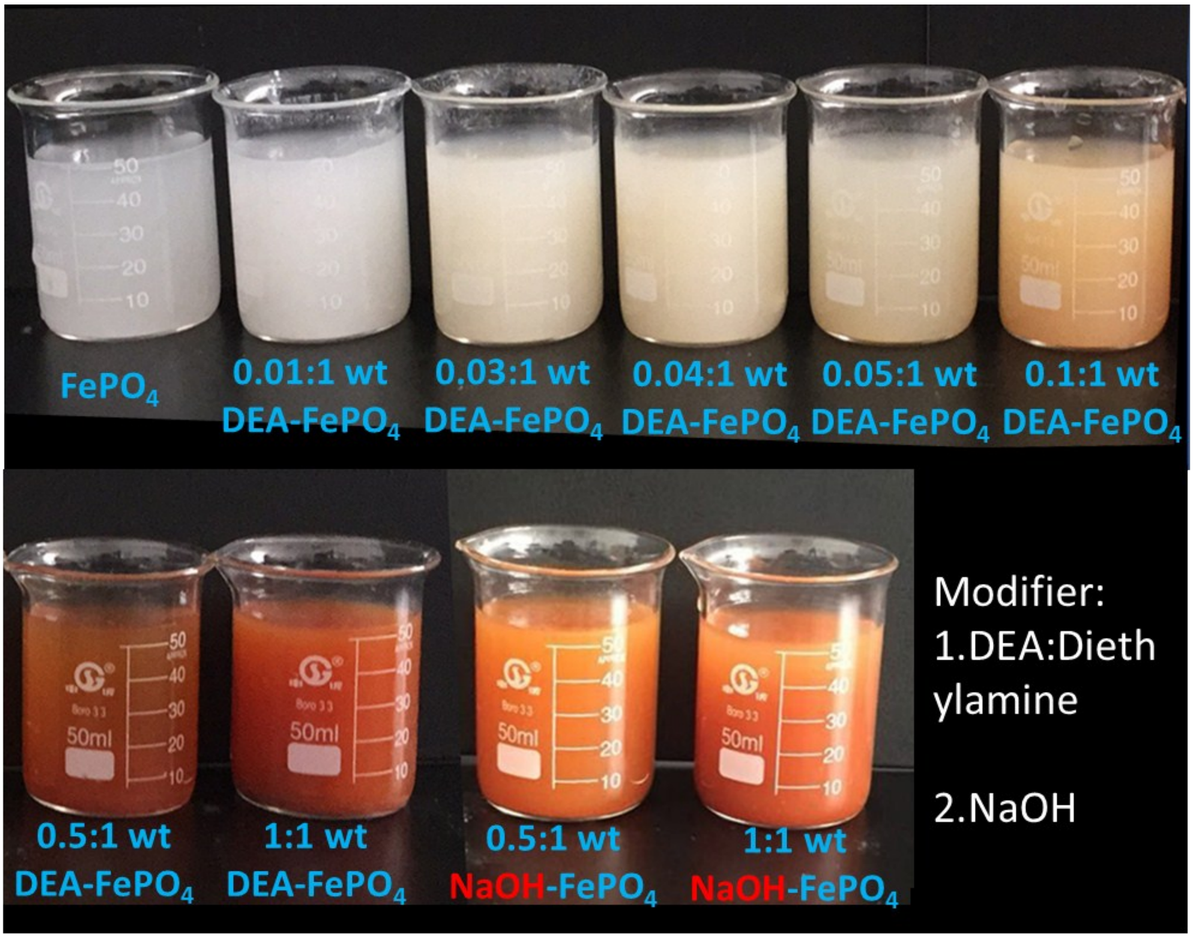

Figure S4 $FePO_4$ suspension with different amount of the diethylamine or the NaOH aqueous solution after 24 h.
